# Special Issue “State-of-the-Art Plant–Virus Interactions in Asia”

**DOI:** 10.3390/v14050864

**Published:** 2022-04-21

**Authors:** Yau-Heiu Hsu

**Affiliations:** 1 Graduate Institute of Biotechnology, National Chung Hsing University, Taichung 402, Taiwan; yhhsu@dragon.nchu.edu.tw; 2 Advanced Plant Biotechnology Center, National Chung Hsing University, Taichung 402, Taiwan

As rivals over the long history of co-evolution, viruses and host plants have each developed specialized strategies and machineries to cope with the rivalry [1,2,3]. Viral genomes have limited coding capacities; thus, they must coerce a plethora of host factors to fulfill the needs in different stages to establish successful infections. In response, plants have evolved various defensive strategies and machineries, both innate and adaptive, general or specialized, against the invading viruses. These interactions between viruses and host plants, in turn, pose evolutionary pressures that lead to the emergence of new viruses or host plant varieties [3]. The comprehension of the interactions between viruses and host plants may facilitate the deeper understanding of the regulation of viral and host gene expression, and the development of more effective methods for the management of plant viral diseases. Furthermore, the knowledge may also be applied to other disciplines, such as horticulture and biotechnology for product improvements.

The interactions between viruses and host plants are complex issues that require scrutiny from different aspects. Some viruses may elicit general defense responses that involve the expression of a common set of proteins, such as pathogenesis-related proteins [4,5], while others may induce the expression of specific host factors which may have positive or negative effects on the infection processes for different viruses [6,7]. These complex interactions may also result in the emergence of new virus isolates and the generation of new host varieties following the mutual adaptations, both naturally and artificially [1]. Over the past few years, many devoted virologists have invested tremendous amounts of energy in dissecting the interactions between viruses and plants at various levels, and have provided excellent and fascinating results that have greatly advanced our knowledge. As more research groups and new technologies join the effort, it is expected that the cross-talks between viruses and plants can be fully comprehended in the foreseeable future.

Asia represents a region of diversity, with environmental and climate conditions ranging from frigid to tropical, thus allowing for the flourish of a wide variety of plants and, of course, viruses. These diverse virus–plant systems provide unique materials for the unraveling of the complex interactions between viruses and plants, as demonstrated by numerous brilliant articles published previously by many Asian research groups. These researches, enforced by the successive studies, have provided insightful clues for the comprehension of the whole interaction network.

In this Special Issue, we have compiled the state-of-the-art researches on the interactions between viruses and plants from world-renowned groups in Asia. The main subjects encompass the following categories. (I) Host factors: Chiu, L. Y. and colleagues identified a novel host factor, lipid transfer protein 1, which facilitates the accumulation of bamboo mosaic virus [8]. On the other hand, several groups provided in-depth analyses of the regulations of antiviral host factors. Chiu, Y. S. and coworkers revealed that fungal F8-culture filtrate may induce the expression of certain defense-related genes which conferred resistance against tomato yellow leaf curl Thailand virus in tomato plants [9]. Sanobar and collaborators compared the functions of HEN1, responsible for small RNA methylation in RNA silencing mechanism, in angiosperm and bryophyte plants under the inhibition of a viral suppressor of RNA silencing, HC-Pro [10]. Diao and colleagues demonstrated that the expression of the antiviral gene, *GmRUN1*, of soybean is modulated by intron-mediated enhancement. In addition to plant factors [11], Zhang and coworkers identified two insect proteins that regulate the replication of rice black-streaked dwarf virus in the insect vector, small brown planthopper [12]. (II) Viral factors: Lee and collaborators demonstrated that the multifunctional P126 protein of odontoglossum ringspot virus unilaterally facilitates the cell-to-cell movement of cymbidium mosaic virus [13]. Chiu, Y. H. and colleagues analyzed the effects of a viral protein, P1/Hc-Pro, on ABA/calcium signaling responses by using high- and low-throughput RNA-Seq approaches [14]. Dai and others identified important viral genome region responsible for the modulation of the direction of leaf curling symptoms and two amino acids in viral C4 protein of certain begomoviruses involved in the modulation of leave curling severity [15]. (iii) New viruses and/or hosts: Yang and colleagues discovered the coinfection of watermelon mosaic virus and a novel polerovirus, provisionally named cotton leaf roll virus, on cotton plants [16]. Lal and collaborators identified a novel geminivirus, likely representing a new genus in the family *Geminiviridae*, in a woody plant, *Fraxinus rhynchophylla*, using high-throughput sequencing technique [17]. Huang and coworkers identified distinctive bamboo mosaic virus isolates on an unreported bamboo species in Vietnam [18]. (iv) Review: Fang and colleagues provided a comprehensive review covering state-of-the-art knowledge on different aspects of the interactions between pepper mottle virus and its host plants [19]. These fascinating research articles and reviews provide the most contemporary information on virus–plant interactions, and highlight the research trends, both for the present and the future.

The great efforts and contributions of the researchers are sincerely appreciated. Sincere gratitude is also extended to the reviewers who have provided critical opinions that helped to significantly improve the contents in this Special Issue. It is anticipated that the outstanding Asian research groups, together with the scientists from different regions in the world, will continue to advance and prosper, and the complexity of the interactions between viruses and plants will eventually be fully comprehended.
